# Hepatic and Splenic Sarcoidosis Without Lymphadenopathy: An Atypical Multisystem Presentation

**DOI:** 10.7759/cureus.101670

**Published:** 2026-01-16

**Authors:** Inês Fiúza M. Rua, João Rodrigues, Sérgio Cabaço, Rodrigo Cavalcanti, Marcel Guerreiro

**Affiliations:** 1 Internal Medicine, Unidade Local de Saúde São José, Lisbon, PRT; 2 Pulmonology, Unidade Local de Saúde São José, Lisbon, PRT; 3 Pathology, Unidade Local de Saúde São José, Lisbon, PRT

**Keywords:** atypical presentation of sarcoidosis, extra pulmonary manifestations of sarcoidosis, hepatic sarcoidosis, hepatosplenomegaly, multisystem sarcoidosis, pulmonary sarcoidosis, sarcoidosis, sarcoidosis without lymphadenopathypathy, splenic sarcoidosis

## Abstract

Sarcoidosis is a multisystem granulomatous disease of unknown etiology characterized by non-caseating granulomas in affected organs. Although pulmonary involvement is most common, extrapulmonary manifestations, particularly hepatic and splenic, may occur in isolation or association. The absence of lymphadenopathy is considered an atypical and uncommon pattern, which may hinder the diagnosis and requires the exclusion of other causes of granulomatous disease. We present a case of a 66-year-old woman with chronic hepatosplenomegaly, mild cytopenias, persistent elevation of cholestatic enzymes, and progressive pulmonary changes without lymphadenopathy. Extensive investigation excluded infectious, autoimmune, and hematologic causes. Liver biopsy confirmed non-necrotizing granulomas consistent with hepatic sarcoidosis. This case highlights the diagnostic challenges posed by atypical sarcoidosis presentations and underscores the importance of considering sarcoidosis in the differential diagnosis of isolated hepatosplenomegaly, even in the absence of lymphadenopathy.

## Introduction

Sarcoidosis is a systemic granulomatous disease of unknown etiology, characterized by the presence of non-caseating granulomas in affected organs. Pulmonary involvement and thoracic lymphadenopathy are the most common manifestations, occurring in over 90% of cases. Extrapulmonary involvement, particularly affecting the liver and spleen, usually occurs in association with pulmonary disease and only rarely as an isolated presentation, typically manifesting as hepatosplenomegaly and/or focal nodular lesions [1-8].

The liver is the third most frequently involved organ, with a prevalence ranging from 5% to 25% among symptomatic patients. Most cases of hepatic sarcoidosis are oligosymptomatic or asymptomatic and are often identified incidentally through laboratory abnormalities, such as elevations in alkaline phosphatase (ALP) and gamma-glutamyl transferase (GGT), or through imaging findings. Splenic involvement is also relatively common in abdominal sarcoidosis, but it is usually associated with lymphadenopathy [2-5,9,10].

The absence of lymphadenopathy, particularly bilateral hilar lymphadenopathy, is considered an atypical and uncommon presentation and may significantly hinder the diagnostic process. In such cases, the diagnosis relies on the histological demonstration of non-caseating granulomas and requires careful exclusion of alternative causes of granulomatous disease, including infectious, malignant, and autoimmune etiologies [1,4,9,10]. Laboratory evaluation may include liver function tests, serum calcium levels, and angiotensin-converting enzyme (ACE) measurement; however, no biomarker is sufficiently sensitive or specific to establish the diagnosis in isolation [1,3,8]. Cross-sectional imaging modalities, such as computed tomography (CT) and magnetic resonance imaging (MRI), are valuable for the characterization of hepatic and splenic involvement and for assessing the extent of systemic disease [1,7,8].

The primary goals of treatment include prevention of disease progression, symptom relief, and reduction of the risk of target organ damage [9,11-13]. While the overall prognosis of sarcoidosis is generally favorable, it largely depends on the extent of organ involvement and the development of complications [5,6,9].

We report a case of pulmonary, hepatic, and splenic sarcoidosis in an asymptomatic patient without lymphadenopathy, with the diagnosis confirmed by liver biopsy.

## Case presentation

A 66-year-old woman was referred to the internal medicine outpatient clinic for evaluation of splenomegaly, mild elevation of liver enzymes, and mild cytopenias detected during routine primary care follow-up. Her medical history was notable for depressive disorder treated with venlafaxine and mirtazapine. She had a low body mass index (17.2 kg/m^2^) without recent weight loss and denied tobacco, alcohol, or illicit drug use. No relevant epidemiological exposures were identified. The patient was asymptomatic.

Physical examination revealed a palpable spleen 4 cm below the left costal margin. No palpable lymphadenopathy, hepatomegaly, or stigmata of chronic liver disease were observed.

Initial laboratory evaluation demonstrated normocytic normochromic anemia (hemoglobin 11.1 g/dL), leukopenia (3.7 × 10^9^/L) with neutropenia (1.9 × 10^9^/L), and normal platelet count. Functional iron deficiency was identified. Peripheral blood smear showed mild anisopoikilocytosis without additional abnormalities. Liver biochemistry revealed elevated GGT (122 U/L) and ALP (157 U/L), with normal bilirubin, transaminases, coagulation parameters, and albumin. Lactate dehydrogenase (LDH) was mildly elevated (274 U/L), with normal haptoglobin. Inflammatory markers showed elevated erythrocyte sedimentation rate (ESR) and low C-reactive protein (CRP). Serum protein electrophoresis revealed polyclonal hypergammaglobulinemia. ACE levels were markedly elevated (238 U/L; reference <52 U/L). Renal function, serum calcium, thyroid function, β2-microglobulin, and urinalysis were normal. Antinuclear antibodies were positive (1:320), with negative extended autoimmune testing. Infectious serologies were negative for acute infection (Table 1). During follow-up, the described abnormalities remained stable (Table 2).

**Table 1:** Investigation for infectious agents HBsAg: hepatitis B surface antigen; HCV: hepatitis C virus; HAV: hepatitis A virus; IgM: immunoglobulin M; IgG: immunoglobulin G; HIV: human immunodeficiency virus; CMV: cytomegalovirus; EBV: Epstein-Barr virus; VCA: viral capsid antigen; EA: early antigen; EBNA: Epstein-Barr nuclear antigen; PCR: polymerase chain reaction; IGRA: interferon-gamma release assay

Laboratory parameter	Result
HBsAg	Negative
Anti-HCV total antibody	Negative
Anti-HAV total antibody	Positive
Anti-HAV IgM	Negative
HIV 1+2 antibody	Negative
CMV IgG	Positive
CMV IgM	Negative
CMV viral load	Negative
EBV - VCA IgG	Positive
EBV - VCA IgM	Negative
EBV - EA IgG	Positive
EBV - EBNA - IgG	Positive
EBV - PCR	Negative
Interferon-gamma release assay IGRA	Negative
Anti-Echinococcus antibody	Negative
Anti-Toxoplasma IgG	Positive
Anti-Toxoplasma IgM	Negative
Parvovirus B19 IgG	Positive
Parvovirus B19 IgM	Negative
Anti-Leishmania (IgG + IgM)	Negative

**Table 2:** Laboratory data during follow-up AST: aspartate aminotransferase; ALT: alanine aminotransferase; ALP: alkaline phosphatase; GGT: gamma-glutamyl transferase; LDH: lactate dehydrogenase; ESR: erythrocyte sedimentation rate; CRP: C-reactive protein; ACE: angiotensin-converting enzyme; TSH: thyroid-stimulating hormone

Laboratory parameter	Day 1	1 week	3 months	6 months	9 months	12 months	Reference range values
Hemoglobin (g/dL)	11.1	11.2	11.3	11.1	10.8	10.5	12.0-15.0
Leukocytes (× 10^9^/L)	3.7	3.6	3.6	3.0	2.6	3.1	4.5-11.0
Neutrophils (× 10^9^/L)	1.9	1.8	1.8	1.4	1.1	1.8	2-8.5
AST (U/L	27	24	22	21	24	20	5-34
ALT (U/L)	17	19	17	13	15	11	0.0-55
ALP (U/L)	157	143	140	136	130	119	40-150
GGT (U/L)	122	111	88	86	74	63	9-36
Total bilirubin (mg/dL)	0.5	0.5	0.4	0.4	0.3	0.4	0.2-1.2
LDH (U/L)	274	260	235	233	247	241	125-220
Creatinine (mg/dL)	0.8	0.7	0.7	0.8	0.7	0.8	0.57-1.11
ESR (mm/h)	65	-	65	71	54	64	<16
Total Protein (g/L)	91.2	83.3	81.4	88.1	86.4	88.0	60-83
CRP (mg/L)	15.6	13.7	21.9	12.4	6.6	27.4	<5.0
ACE (U/L)	-	238	-	240	272	-	8-52
Serum Calcium (mg/dL)	9.1	9.1	8.9	9.4	9.1	9.5	8.8-10.2
Thyroid-Stimulating Hormone (µUI/mL)	3.5	-	-	4.1	-	-	0.27-4.2
β2 -microglobulin (mg/L)	-	2.86	-	-	-	-	<3.0
Urinalysis - pH	7	-	-	-	-	-	5-8
Urinalysis - Leukocytes (/µL)	10	-	-	-	-	-	<11
Urinalysis - Erythrocytes (/µL)	22	-	-	-	-	-	<17
Urinalysis - Squamous Epithelial Cells (/µL)	6	-	-	-	-	-	<5
Urinalysis - Transitional Epithelial Cells (/µL)	2	-	-	-	-	-	-

**Figure 1:**
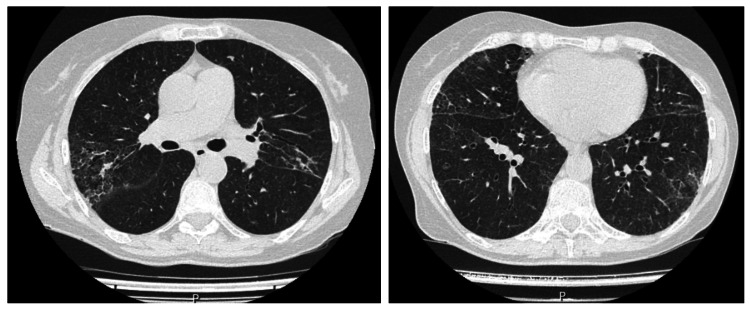
Thoracic computed tomography scan showing pulmonary findings suggestive of interstitial involvement Findings include interlobular septal thickening, mild subpleural reticulation, fibrotic changes, and traction bronchiectasis, predominantly affecting the middle lobe, lingula, and bilateral basal regions, without evidence of abdominal lymphadenopathy.

**Figure 2:**
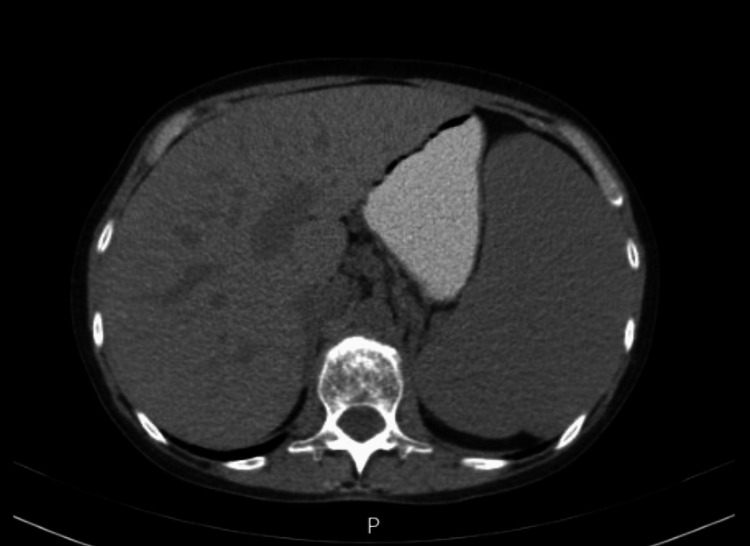
Abdominal CT scan demonstrating hepatosplenomegaly Diffuse enlargement of the liver with caudate lobe hypertrophy and homogeneous splenomegaly are observed, without evidence of abdominal lymphadenopathy.

Abdominal ultrasound confirmed homogeneous splenomegaly (17 × 10.4 cm) with engorgement of splenic hilar vessels. Transthoracic echocardiography and Doppler ultrasound of the hepatosplenic circulation excluded congestive etiologies. Thoracoabdominal-pelvic CT revealed pulmonary interstitial abnormalities characterized by interlobular septal thickening and subpleural reticulation, predominantly in the middle lobe, lingula, and basal regions, with fibrotic changes and traction bronchiectasis, consistent with progression of previously identified ground-glass and consolidative lesions (Figure 1). Global hepatomegaly with caudate lobe hypertrophy and splenomegaly were observed, without lymphadenopathy (Figure 2).

**Figure 3:**
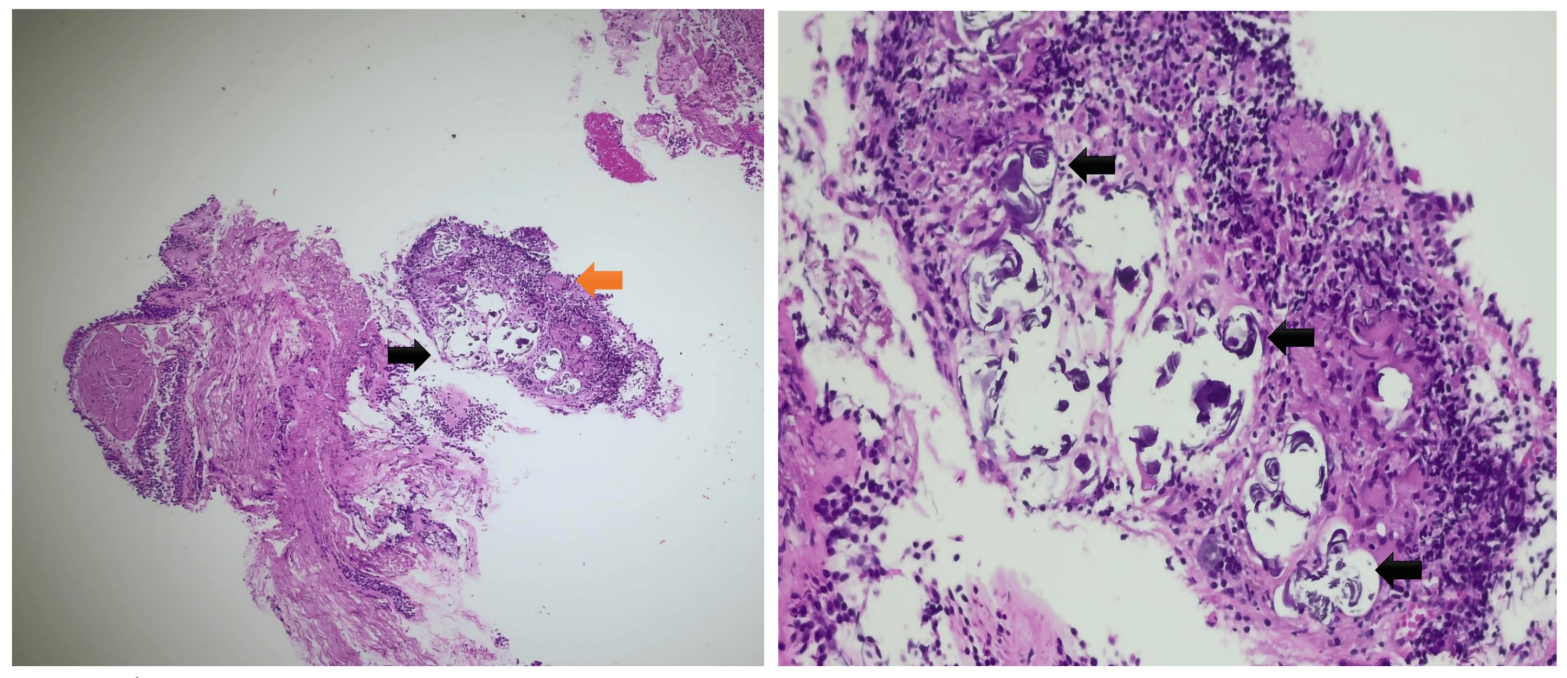
Bronchial wall biopsy specimens Left: Aggregates of multinucleated giant cells (orange arrow) containing Schaumann bodies (black arrow), without well-formed epithelioid granulomas (hematoxylin and eosin stain, ×50). Right: Higher magnification highlighting intracytoplasmic Schaumann bodies within multinucleated giant cells (black arrows) (hematoxylin and eosin stain, ×200).

Bronchoscopy with bronchial and bronchoalveolar lavage was negative for microbiological, mycobacterial, and fungal studies. Lymphocyte subset analysis revealed a CD4/CD8 ratio of 0.57. Bronchial biopsies demonstrated fibrosis and mild inflammatory infiltrate with small aggregates of multinucleated giant cells containing Schaumann bodies, findings suggestive but not diagnostic of sarcoidosis (Figure 3).

**Figure 4:**
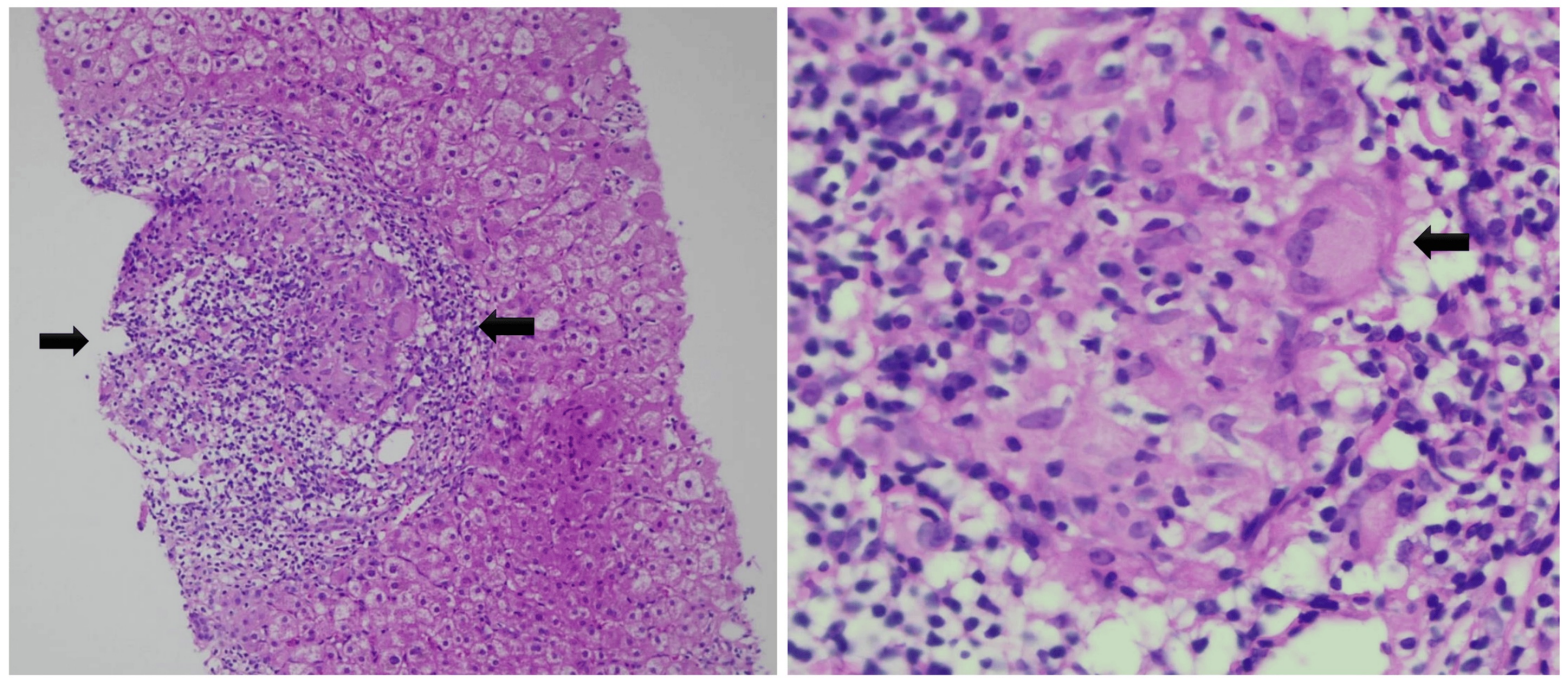
Liver biopsy histopathology Left: Non-necrotizing granuloma (arrow) with associated inflammatory infiltrate (hematoxylin and eosin stain, ×50). Right: Granuloma with prominent multinucleated giant cells (arrow) (hematoxylin and eosin stain, ×200).

Given the high clinical suspicion, a percutaneous liver biopsy was performed. Histopathological examination revealed non-necrotizing granulomas of moderate size in portal and lobular regions, with associated lymphocytic infiltrate and intralesional fibrosis. Special stains for microorganisms (Ziehl-Neelsen, periodic acid-Schiff (PAS), and Grocott) were negative (Figure 4).

Systemic corticosteroid therapy with prednisolone 30 mg/day was initiated, and the patient was referred to pulmonology. After one year of follow-up, she remained asymptomatic, with improvement in bicytopenia, partial improvement of liver enzyme abnormalities, and normalization of ACE levels under corticosteroid therapy (32 U/L). Due to corticosteroid-induced osteoporosis with multiple vertebral compression fractures, steroid-sparing therapy with methotrexate was introduced, and prednisolone dosage was reduced.

## Discussion

Sarcoidosis with hepatic and splenic involvement in the absence of lymphadenopathy is an uncommon presentation and is frequently misdiagnosed as malignant infiltration, granulomatous infection, or autoimmune disease [2,11,13,14]. Given the presence of splenomegaly and cytopenias, hematologic malignancy, particularly lymphoma, was a major initial diagnostic consideration. However, the absence of lymphadenopathy or B symptoms, stable mild cytopenias over time, normal β2-microglobulin levels, and the lack of malignant features on bronchial and liver histopathology argued against this diagnosis. In the present case, the absence of symptoms, persistent splenomegaly, mild cytopenias, and chronic elevation of ALP and GGT prompted investigation for chronic infiltrative disease.

ACE levels are elevated in approximately 60% of patients and, although nonspecific, supported the diagnostic hypothesis [12,13]. Splenomegaly may occur in up to 40-60% of patients with abdominal sarcoidosis, but is usually associated with lymphadenopathy; its absence represents an atypical variant described mainly in small series [2,10,13,14].

Pulmonary involvement remains the main determinant of morbidity and mortality and may occur without hilar lymphadenopathy [2,10,14,15]. In this case, CT demonstrated chronic inflammatory changes and traction bronchiectasis compatible with advanced and fibrotic sarcoidosis.

Schaumann bodies, identified within multinucleated giant cells, represent morphological markers of mature granulomas and chronic inflammation. Although characteristic, they are not pathognomonic and may be observed in other granulomatous diseases, emphasizing the need for exclusion of alternative etiologies [11,16].

Hepatic involvement is often asymptomatic and identified through cholestatic liver enzyme abnormalities or hepatomegaly [9,10-13]. In this case, the presence of non-caseating granulomas on liver histology, together with exclusion of infectious agents, was diagnostic of sarcoidosis.

In asymptomatic patients without evidence of organ dysfunction, observation may be appropriate, as subclinical disease can be self-limited. Treatment is reserved for symptomatic patients or those at risk of organ damage. Systemic corticosteroids are first-line therapy, with more than 50% of patients requiring prolonged treatment. Steroid-sparing agents such as methotrexate or biologic therapies may be used in refractory or chronic cases [3-5,10,17]. Given the presence of progressive pulmonary disease and hepatic and splenic involvement, targeted therapy was initiated in accordance with international recommendations [9,11-13].

As a single-case report, this observation is inherently limited in its generalizability; however, it highlights an uncommon presentation of sarcoidosis that may aid clinicians in recognizing similar diagnostic patterns in clinical practice.

## Conclusions

This case illustrates an atypical presentation of multisystem sarcoidosis with hepatic, splenic, and pulmonary involvement in the absence of lymphadenopathy. The lack of lymphadenopathy delayed diagnosis and necessitated an extensive diagnostic workup. Liver biopsy was essential for definitive diagnosis. Recognition of such uncommon presentations is crucial for timely diagnosis and appropriate management.
